# Molecular characterization and biofilm formation ability of *Enterococcus faecium* and *Enterococcus faecalis* bloodstream isolates from a Chinese tertiary hospital in Beijing

**DOI:** 10.1007/s10123-023-00441-2

**Published:** 2023-11-06

**Authors:** Jing-xian Yang, Cun-wei Liu, Fu-wei Wu, Ling Zhu, Guo-wei Liang

**Affiliations:** Department of Clinical Laboratory, Aerospace Center Hospital, Beijing, 100049 China

**Keywords:** *Enterococcus faecium*, *Enterococcus faecalis*, Vancomycin-resistant, Multilocus sequence typing, Virulence, Biofilm

## Abstract

To investigate the molecular characteristics and biofilm-forming ability of 116 *Enterococcus faecium* (*Efm*) and 72 *Enterococcus faecalis* (*Efs*) isolates obtained from patients with bloodstream infections (BSI) at a Chinese hospital between July 2011 and March 2018. The presence of glycopeptide resistance genes and five virulence genes (*esp*, *gelE*, *asa1*, *hyl*, and *cylA*) was screened using two multiplex PCR. MLST was used to assess the clonality. Crystal violet staining was used to detect biofilms. Vancomycin resistance was detected in 30.1% of *Efm* and 2.8% of *Efs* isolates, respectively. All VRE strains carried the *vanA* gene. The *esp*, *gelE*, *asa1*, and *cylA* genes in 72 *Efs* strains were detected at 62.5%, 84.7%, 84.7%, and 69.4%, respectively. Among the 116 *Efm* isolates, 74.1% and 25.8% carried *esp* and *hyl*, respectively. The *esp* gene was significantly associated with vancomycin-resistant *Efm* (VREfm) compared to vancomycin-susceptible *Efm* (VSEfm). In total, 91.7% of *Efs* and 20.0% of *Efm* produced biofilms. Twenty-six STs were identified among the 72 *Efs* isolates, with ST4 (29.2%) being the predominant. In total, 116 *Efm* strains were grouped into 26 STs, with ST78 (46.6%) being the predominant. Both VREfm (41.7%) and VSEfm (48.8%) were dominant in ST78. There is no clear evidence suggesting that some STs are associated with vancomycin resistance or biofilm formation. Both *Efm* and *Efs* BSI isolates showed a polyclonal pattern with a dominant clone and many unique types, implying the coexistence of clonal dissemination and an influx of new clones. The horizontal transmission of resistance genes may play a more important role in VREfm prevalence than clonal expansion.

**Supplementary Information:**

The online version contains supplementary material available at 10.1007/s10123-023-00441-2.

## Introduction

*Enterococcus* is a large genus of gram-positive bacteria containing more than 50 species, the most common of which are *Enterococcus faecium* (*Efm*) and *Enterococcus faecalis* (*Efs*) when isolated from clinical specimens of hospitalized patients (Freitas et al. 2021; Schell et al. 2020). Data from the SENTRY Antimicrobial Surveillance Program show that *Enterococcus* ranks fourth in North America (10.2%) and fifth in Europe (7.2%) as the pathogen that causes bloodstream infections (BSI) (Deshpande et al. 2007). BSI caused by *Enterococcus* species are associated with substantial mortality, particularly in hospitalized populations and those with multiple comorbidities. It is clinically difficult to treat because of its natural (cephalosporin) or acquired resistance (ampicillin, vancomycin) (Contreras et al. 2022).

There were significant differences in pathogenicity and drug resistance between *Efm* and *Efs*. *Efm* is more resistant than *Efs*, whereas *Efs* is more prone to biofilm formation (Zheng et al. 2018). Moreover, the ratio of *Efs* to *Efm* causing diseases has changed greatly over the past few decades, from 10:1 before 1990 to 5:1 in the 1990s, 3:1 in the early 2000s, and now to 1.5:1 or even 1:1 (Freitas et al. 2021). However, the reason for this remains unclear. At our hospital, this ratio was inverted to 1:1.6. According to the 2022 China Antimicrobial Surveillance Network (CHINET) reports, *Efm* and *Efs* ranked sixth (4.3%) and tenth (2.3%), respectively, among 50,209 blood isolates (www.chinets.com). In our hospital, *Efm* and *Efs* ranked fifth (6.2%) and ninth (3.9%), respectively, for BSI in 2022.

In addition, the rapid worldwide dissemination of vancomycin-resistant *Enterococcus* (VRE) has become a significant global burden on healthcare systems. The Centers for Disease Control and Prevention (CDC) categorized VRE as “Serious Threats” in its 2019 Antibiotic Resistance Threats Report and estimated that it could cause 54,500 infections among hospitalized patients and 5400 deaths per year in the USA (Center for Disease Control and Prevention 2019). The rates of VRE are highly variable in different regions (< 1–86.7%), with increasing trends in some countries (Freitas et al. 2021). Therefore, identifying and controlling the spread of *Enterococcus* spp. in healthcare settings is crucial. In China, the prevalence of VRE has remained very low (< 3%) in recent years according to reports from the China Antimicrobial Resistance Surveillance System (CARSS) (http://www.carss.cn). However, our hospital has had an extremely high VRE rate over the last 10 years, fluctuating between 30 and 40%. The causes and factors contributing to the higher prevalence of VRE in our hospital remain unknown.

To date, several studies (Contreras et al. 2022; Ryan et al. 2015; Sun et al. 2019; Zhou et al. 2020) have been conducted to understand the molecular characteristics of resistance, virulence, and clonality in local VRE strains, including those of our group (Yang et al. 2015). Most studies involving multilocus sequence typing (MLST) analysis have focused only on VRE strains; vancomycin-susceptible *Enterococcus* (VSE) strains in the corresponding region have rarely been monitored. In fact, understanding the respective sequence type (ST) distribution of VRE and VSE strains could help us understand whether certain STs are more prone to developing glycopeptide resistance and clonal transmission. In our previous study (Yang et al. 2015), we identified 12 STs in 69 vancomycin-resistant *Enterococcus faecium* (VREfm) strains, with ST78 accounting for the majority (42.0%). However, it was unclear whether clonal spread was present in ST78 isolates that were already resistant or whether ST78 itself was more prevalent, regardless of resistance or susceptibility.

To answer this question, we collected 116 *Efm* (36VRE + 80VSE) and 72 *Efs* (2VRE + 70VSE) isolates from the positive blood culture specimens at the Aerospace Center Hospital between July 2011 and March 2018 to comprehensively and intensively investigate their molecular characterization and biofilm formation ability.

## Materials and methods

### Bacterial isolates

In total, 116 *Efm* and 72 *Efs* isolates were collected from positive blood culture specimens at the Aerospace Center Hospital (Beijing, China) between July 2011 and March 2018. The hospital is a tertiary teaching hospital with 1200 beds. Consecutive enterococcal isolates and one colony per patient were collected. All strains were kept frozen at –80℃. All isolates were confirmed using matrix-assisted laser desorption/ionization time-of-flight (MALDI-TOF) mass spectrometry (MS) (Vitek MS, bioMerieux, France).

### Antimicrobial susceptibility testing

Antimicrobial susceptibility testing was performed using GP67 cards from the Vitek 2 Compact (bioMérieux, France). The disc diffusion method was performed twice to confirm the presence of VRE, according to the Clinical and Laboratory Standards Institute guidelines (Clinical and Laboratory Standards Institute 2020). Ten antimicrobial agents were tested: penicillin, ampicillin, high-level gentamicin, levofloxacin, ciprofloxacin, erythromycin, tetracycline, vancomycin, linezolid, and tigecycline. The ATCC 29212 strain of *Efs* and the ATCC 29213 strain of *S. aureus* were used as quality controls. The results of antimicrobial susceptibility testing were interpreted according to the Clinical and Laboratory Standards Institute 2020 and European Committee on Antimicrobial Susceptibility Testing (EUCAST) 2020 breakpoint criteria when available (Clinical and Laboratory Standards Institute 2020; European Committee on Antimicrobial Susceptibility Testing (EUCAST) 2020) .

### Detection of glycopeptide resistance genes and virulence genes

Bacterial DNA was extracted using the Bacterial Genomic DNA Extraction Kit D1600 (Solarbio Science and Technology, Beijing, China). The presence of the glycopeptide resistance genes *vanA* and *vanB* was detected using a multiplex PCR assay as previously described (Yang et al. 2015). Five potential virulence genes (*esp*, *gelE*, *asa1*, *hyl*, and *cylA*) in the enterococci were simultaneously analyzed using a previously published multiplex PCR method (Yang et al. 2015).

### Multilocus sequence typing (MLST)

MLST of *Efm* and *Efs* strains was conducted using previously published methods (Yang et al. 2015). STs were determined by amplifying and sequencing seven housekeeping genes for *Efm* (*atpA*, *ddl*, *gdh*, *purK*, *gyd*, *pstS*, and *adk*) and *Efs* (*gdh*, *gyd*, *pstS*, *gki*, *aroE*, *xpt*, and *yiqL*) according to the MLST databases (https://pubmlst.org/organisms/enterococcus-faecium and https://pubmlst.org/organisms/enterococcus-faecalis). The minimum spanning tree (MST) analysis was obtained using the BURST algorithm for related STs between different backgrounds by means of BioNumerics 7.6 software (Applied Maths, Belgium). By BURST algorithm, strains were divided into “clusters” according to their allelic profiles. The same cluster was defined as a set of STs that matched more than six of the seven loci.

### Biofilm formation assay

According to the literature (Stepanović et al. 2007) and local laboratory conditions, a biofilm infection model of *Enterococcus* was established in vitro using a 96-well plate. Crystal violet staining was used to detect the biofilm formation ability. Briefly, (1) a single fresh colony was taken and placed in 10 mL tryptic soy broth (TSB) and shaken overnight at 37℃; (2) 2 µL of the above bacterial solution and 198 µL TSB were added to the 96-well plate and incubated overnight at 37℃, repeating each strain in triplicate. ATCC29212 strain of *Efs* was used as a positive control and sterile TSB as a negative control; (3) the plate was washed three times with 200 µL PBS, fixed with 200 µL anhydrous ethanol for 30 min, stained with 0.5% crystal violet for 10 min, and then washed three times with 200 µL PBS; (4) the crystal violet was dissolved in 100 µL anhydrous ethanol for 5 min and the optical density (OD) was measured at 595 nm. Each well was measured three times, and the average of nine measurements was calculated for each bacterial strain.

The criteria for interpreting biofilm assays were based on the literature (El-Zamkan and Mohamed 2021), six negative control wells were included in each batch, and each well was measured three times, giving a total of 18 results. The mean of the negative control plus three standard deviations was used as the cutoff value (Dc). The abilities of strain to form biofilm were categorized as none (OD ≤ Dc), weak (Dc < OD ≤ 2 Dc), moderate (2Dc < OD ≤ 4 Dc), and strong (4Dc < OD).

## Statistical analysis

Statistical analyses were performed using the SPSS software (version 16.0, SPSS Inc.). Differences in the prevalence of the tested features in *Efm* and *Efs* strains were assessed using chi-squared tests. Chi-square values were corrected when the number of isolates was less than 40, or at least one expectation was between 1 and 5. A *P*-value of < 0.05 was considered as statistically significant.

## Results

### Bacterial distribution

Between July 2011 and March 2018, 208 enterococcal isolates were screened from patients with BSI. Among them, *Efm* (*n* = 116, 55.8%) and *Efs* (*n* = 72, 34.6%) were the most common, and the remaining 20 (20/208, 9.6%) were other enterococci such as *E. gallinarum*. All *Efm* and *Efs* strains identified and distributed across the various departments were included in this study. *Efm* and *Efs* were most widely and evenly distributed in internal medicine, surgical, and ICU wards with some exceptions (Table 1). In the Department of Hematology, 14 (14/116, 12.1%) *Efm* were detected, but without a single case of *Efs* (*P* < 0.05)*.* Interestingly, more *Efm* was found than *Efs* in most wards, whereas in neurology wards, *Efs* were detected in significantly higher numbers than *Efm* (*P* < 0.05)*.* This discrepancy in bacterial distribution among different wards indicates that pathogenic bacteria are distributed differently among different wards, even within the same hospital. Therefore, localized data monitoring should be conducted for each specific ward.

**Table 1:** Department distribution of 116 *Efm* and 72 *Efs* strains

Departments	*Efm* (*n* = 116)		*Efs* (*n* = 72)		*χ*^2^	*P* value
	*n*	%	*n*	%		
Internal medicine	69	57.5	33	45.8	3.3	0.068
Neurology	2	1.7	13	16.7	14.0	< 0.001*
Surgery	15	12.9	14	20.8	1.4	0.229
Intensive care unit	11	9.5	7	9.7	0.003	0.957
Hematology	14	12.1	0	0	7.7	0.005*
Emergency	5	4.3	2	2.8	0.02	0.886
Gynecology and obstetrics	0	0	3	4.2	2.6	0.106

* P < 0.05, statistically significant

### Antimicrobial susceptibility

Resistance rates of 116 *Efm* and 72 *Efs* to the antimicrobial agents are shown in Table 2. In general, *Efm* showed significantly higher resistance rates than *Efs* except for erythromycin, high-level gentamicin, and tetracycline. Ampicillin resistance was observed in 78.4% (91/116) and 30.6% (22/72) of *Efm* and *Efs* isolates, respectively (*P* < 0.05). Vancomycin resistance was detected in 30.1% (36/116) and 2.8% (2/72) of *Efm* and *Efs* isolates, respectively (*P* < 0.05). Only one strain of *Efs* was resistant to linezolid, and no resistance to tigecycline was observed. For *Efm*, more than 45% of the isolates were resistant to penicillin, ampicillin, high-level gentamicin, levofloxacin, erythromycin, and tetracycline. For *Efs*, however, more than 55% of the isolates were resistant to high-level gentamicin, levofloxacin, erythromycin, and tetracycline.

**Table 2:** Resistance rates (%) of 116 *Efm* and 72 *Efs* strains to 10 antimicrobial agents

Antibacterial agents	*Efm* (%)	*Efs* (%)	χ^2^	*P* value
Penicillin	85.3	37.5	46.0	< 0.001*
Ampicillin	78.4	30.6	42.5	< 0.001*
High-level gentamicin	49.1	63.9	3.9	0.048*
Levofloxacin	77.6	55.6	10.1	0.001*
Ciprofloxacin	77.6	55.6	10.1	0.001*
Erythromycin	82.8	72.2	2.9	0.086
Tetracycline	46.6	77.8	17.8	< 0.001*
Vancomycin	31.0	2.8	22.0	< 0.001*
Linezolid	0.0	1.4	1.6	0.203
Tigecycline	0.0	0.0	-	-

* P < 0.05, statistically significant

### VRE prevalence and glycopeptide resistance genes

Of the 72 *Efs* strains tested, only two (2.8%) were identified as VRE, and both were isolated in 2012 from the neurology department. This extremely low resistance rate is consistent with the CARSS data (0.2%). In *Efm*, the prevalence of VRE was alarmingly high at 30.1% (36/116), significantly exceeding the national average rate of 1.2% and the average rate of 9.9% in Beijing (data from CARSS). All these VRE strains, regardless *Efm* or *Efs*, carried the *vanA* gene without any exceptions, which is in agreement with other studies (Egan et al. 2022; Liu et al. 2022; Sun et al. 2019), suggesting that *vanA* was still the dominant resistance determinant in enterococci in Beijing.

### Virulence genes and biofilm formation ability

**Fig. 1:**
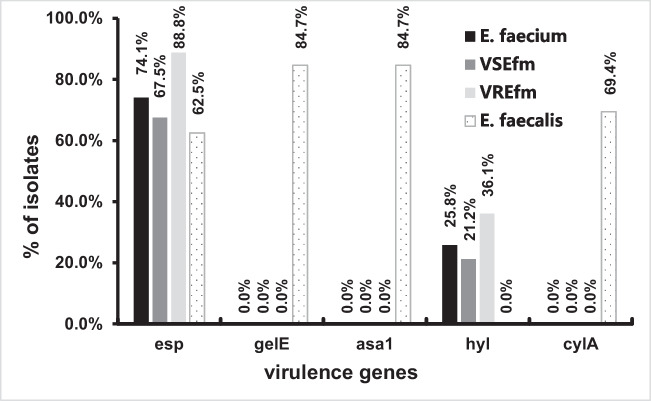
Prevalence of five virulence genes distributed in 116 *Efm*, 80 vancomycin-susceptible *Efm* (VSEfm), 36 vancomycin-resistant *Efm* (VREfm) and 72 *Efs* strains

Among the 116 *Efm* isolates, 74.1% (86/116) and 25.8% (30/116) carried *esp* and *hyl*, respectively (Fig. 1). None harbored the *asa1*, *gelE*, or *cylA* genes. In 72 *Efs* strains, *esp*, *gelE*, *asa1*, and *cylA* were detected at incidence of 62.5% (45/72), 84.7% (61/72), 84.7% (61/72), and 69.4% (50/72), respectively, whereas *hyl* was not detected in any strain. Furthermore, 48.6% (35/72) of the isolates showed the coexistence of all four virulence genes. There was no statistically significant difference in the carriage of *esp* between *Efm* and *Efs* (*χ*^2^ = 2.9, *P* = 0.09)*.*

It is worth mentioning that the prevalence of *esp* in VRE (32/36, 88.8%) of *Efm* was higher than that of VSE (54/80, 67.5%), with a significant difference (*χ*^2^ = 5.9, *P* = 0.015). Meanwhile, the prevalence of *hyl* in VRE (13/36, 36.1%) of *Efm* was also higher than that of VSE (17/80, 21.2%); however, the difference was not statistically significant (*χ*^2^ = 2.9, *P* = 0.09). These results suggest that *esp* is associated with vancomycin resistance, whereas *hyl* may be less related. Most of these features of virulence gene distribution are similar to those reported in studies from other countries, such as India (Rao et al. 2021) and Turkey (Erdem et al. 2020).

Of the 72 *Efs* strains examined, only six (8.3%) did not demonstrate biofilm formation. The remaining 66 (91.7%) strains showed significant biofilm formation, which were categorized as weak, moderate, or strong, accounting for 20.8% (15/72), 43.1% (31/72), and 27.8% (20/72), respectively. This is strikingly different from *Efm*, where only 22 (20.0%) of the 116 strains produced biofilms, of which 21 were weak and only one was strong, which is consistent with the results of other studies (El-Zamkan and Mohamed 2021; Zheng et al. 2018). The original biofilm-forming ability data for each strain are presented in Tables S1.

### Relationship between biofilm and virulence genes in 72 Efs strains

The high virulence gene carriage (62.5–84.7%) and biofilm formation capacity (91.7%) of the 72 *Efs* strains resulted in a low number of strains in the virulence factor-negative and no biofilm formation groups, which prevented a statistical comparison between the groups. The data illustrated in Table 3 reveal that most (41.0–57.8%) of the four virulence gene-positive strains showed moderate biofilm formation, while three of the virulence gene-negative strains were predominantly (44.4–50.0%) strong biofilm producers, with the exception of the *gelE*-negative strains (54.5%), which produced primarily moderate-level biofilms.

**Table 3:** Relationship between biofilm formation capacity and virulence genes of 72 *Efs* strains

Virulence genes (*n*)	Biofilm formation capacity [*n* (%)]			
	None	Weak	Moderate	Strong
*esp* + (45)	2 (4.4)	9 (20.0)	26 (57.8)	8 (17.8)
*esp*- (27)	4 (14.8)	6 (22.2)	5 (18.5)	12 (44.4)
*gelE* + (61)	6 (9.8)	12 (19.7)	25 (41.0)	18 (29.5)
*gelE*- (11)	0 (0.0)	3 (27.3)	6 (54.5)	2 (18.2)
*asa1* + (61)	5 (8.2)	12 (19.7)	29 (47.5)	15 (24.6)
*asa1*- (11)	1 (9.1)	3 (27.3)	2 (18.2)	5 (45.5)
*cylA* + (50)	4 (8.0)	9 (18.0)	28 (56.0)	9 (18.0)
*cylA*- (22)	2 (9.1)	6 (27.3)	3 (13.6)	11 (50.0)

### Relationship between biofilm and glycopeptide resistance in 116 Efm stains

As mentioned above, 22 (20.0%) isolates formed biofilms in 116 *Efm* strains, of which eight were VRE and 14 were VSE. In other words, biofilm was produced by 22.2% (8/36) of VRE and 17.5% (14/80) of VSE, and the difference was not statistically significant (*χ*^2^ = 0.4, *P* = 0.55). In addition, the only strong biofilm-forming strain was vancomycin susceptible. Our findings indicate that vancomycin resistance is not associated with biofilm formation.

### Molecular typing by MLST

**Fig. 2:**
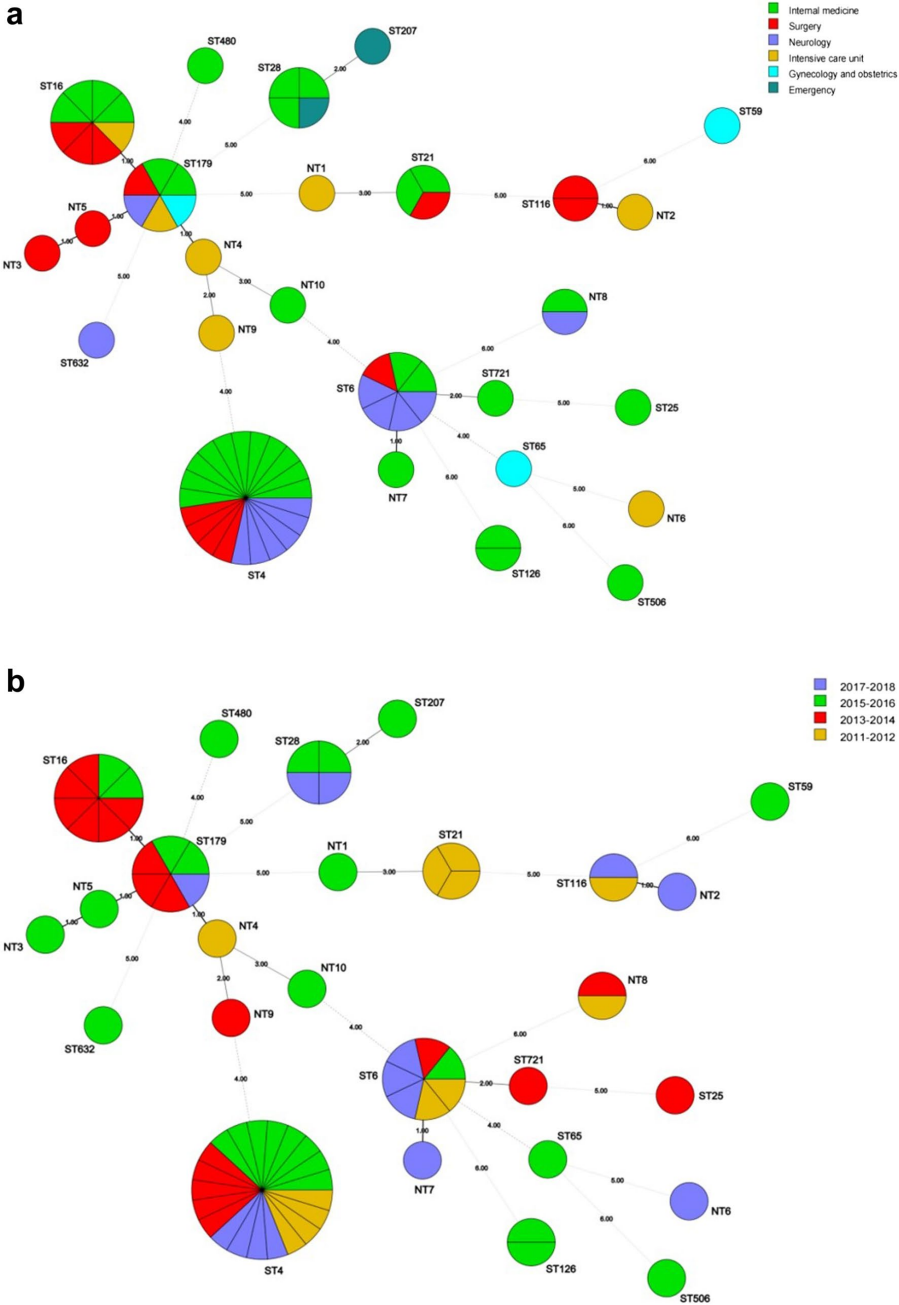
Minimum spanning tree analysis based on MLST data from 72 *Enterococcus faecalis* isolates*.* Each ST is presented as a circle, and the size of the circle indicates the number of strains belonging to that particular ST. Numbers on the lines between each circle indicate the number of different alleles. The larger the numbers, the more distant the genetic relationship. Each circle is labeled with the corresponding ST. Different groups of strains are distinguished by different colors. The strains have been divided into six groups according to departmental distribution (**A**) and four groups according to year of isolation (**B**)

Among the 72 *Efs* isolates analyzed, 26 distinct STs were identified, with ST4 (*n* = 21, 29.2%) being the most predominant, followed by ST16 (*n* = 8, 11.1%), ST6 (*n* = 7, 9.7%), and ST179 (*n* = 6, 8.3%). The remaining 22 STs included 12 known STs (ST28, ST21, ST116, ST126, ST25, ST59, ST65, ST207, ST480, ST506, ST632, and ST721) and ten new ones (NT1–NT10), and each of those covered no more than four strains. Both VRE strains belonged to ST4. Figure 2 shows the distribution of STs in different departments and years 2011–2018. After MST analysis, out of the 16 known ST types, only ST16 and ST179 belonged to the same cluster, that is, ST179 (5/1/1/3/7/1/6) was a single-locus variant (SLV) of ST16 (5/1/1/3/7/7/6), while the remaining 14 STs were singletons, independent of each other and distantly related, suggesting that the emergence and spread of *Efs* in our hospital is marked by genetic heterogeneity, with clonal spread occurring only for ST4.

**Fig. 3:**
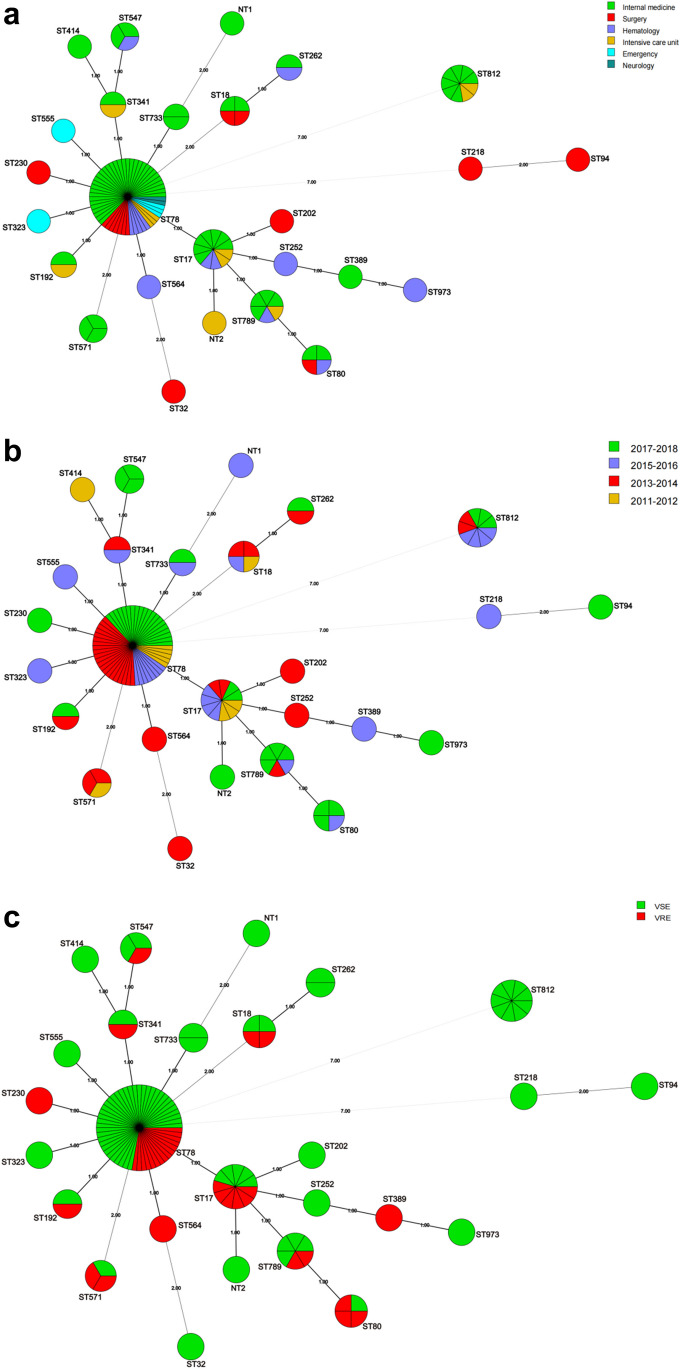
Minimum spanning tree analysis based on MLST data from 116 *Enterococcus faecium* isolates. The strains were divided into six groups according to departmental distribution (**A**), four groups according to year of isolation (**B**), and two groups according to vancomycin resistance (**C**)

For *Efm*, the 116 strains were grouped into 26 STs. Of these, ST78 (*n* = 54, 46.6%) was the most common, accounting for approximately half of all the strains, followed by ST17 (*n* = 11, 9.5%), ST812 (*n* = 9, 7.8%), and ST789 (*n* = 6, 5.2%). Additionally, ST18 and ST80 each accounted for four strains (3.4%), while ST547 and ST571 each accounted for three strains (2.6%). Finally, other STs, including ST192, ST262, ST341, ST733, ST32, ST94, ST202, ST218, ST230, ST252, ST323, ST389, ST414, ST555, ST564, and ST973, and two new STs (NT1–NT2), covered only two or one strains. Figure 3 shows the distribution of STs among the 116 *Efm* bacteria examined in this study. According to MST analysis, ST18 and ST262 belong to the same cluster; ST812, ST571, ST32, ST94, ST218, and NT1 are singletons; and the remaining 18 STs belong to the same cluster, formerly known as the clonal complex (CC) 17 (Yang et al. 2015). This is in sharp contrast to the heterogeneity of *Efs* strains, where most STs in *Efm* strains tended to be more closely related, despite the number of ST types in both being 26.

Overall, each ST was almost evenly distributed across different wards and years, with no obvious relationship between any particular ST and ward (Fig. 2A and 3A) or year of infection (Fig. 2B and 3B), regardless of *Efm* or *Efs*. The only possible patterns were that three *Efs* strains belonging to ST21 were only detected between 2011 and 2012, three *Efm* strains of ST571 were only detected before 2014, and three *Efm* strains of ST547 were only detected between 2017 and 2018.

### Relationship between MLST and glycopeptide resistance in 116 Efm stains

As shown in Fig. 3C, 12 different STs were identified in 36 VREfm strains and 23 in 80 vancomycin-susceptible *Enterococcus faecium* (VSEfm) strains. Both were most common in ST78, with prevalence rates of 41.7% (15/36) and 48.8% (39/80), respectively (*χ*^2^ = 0.5, *P* = 0.48). This result suggests that ST78 is widely present in our hospital, regardless of vancomycin resistance. It is possible that the clonal spread of already vancomycin-resistant ST78 was not the main cause of the outbreak. As stated above, although there is a large variety of STs, with the exception of ST78, ST17, ST812, and ST789, the remaining 22 STs are all less than four cases; therefore, even though some STs only occur in VREfm (ST230, ST564, and ST389) and some only in VSEfm (ST733, ST262, etc.), the number is too small to be representative.

Notably, all nine strains of ST812 were vancomycin-susceptible. These nine strains were isolated from different wards in different years. ST812 differed from ST78 in all the seven housekeeping genes tested. However, whether ST812 is not prone to produce vancomycin resistance requires further investigation.

### Relationship between MLST and biofilm

In the 116 *Efm* strains, MLST analysis showed that the 22 biofilm-positive isolates could be classified into ten STs, including 12 ST78 (54.5%), two ST789 (9.1%), and one each of ST17, ST18, ST94, ST230, ST341, ST555, ST733, and ST812. The 94 biofilm-negative isolates were dominated by ST78 (44.7%, 42/94), with a small number of the remaining 22 STs. Of the 72 *Efs* strains, six (8.3%) biofilm-negative strains belonged to five different STs. The present results suggest that there is no definite evidence that some STs are more significantly associated with biofilm formation than others in both *Efm* and *Efs*.

## Discussion

This was a single-center, cross-sectional and retrospective study on the molecular characterization and biofilm formation ability of 116 *Efm* and 72 *Efs* isolated from blood samples at a Chinese tertiary hospital in Beijing. The comprehensive genomic and phenotypic analyses led to several conclusions. First, the resistance rates to ampicillin (78.4% vs. 30.6%) and vancomycin (30.1% vs. 2.8%) were significantly higher in *Efm* isolates compared to *Efs* isolates. Second, *Efs* isolates possessed more virulence traits than *Efm* isolates, and the percentage of biofilm-forming isolates (91.7% vs. 22.0%) was higher in *Efs* isolates than in *Efm* isolates. Third, a total of 26 STs were detected for both *Efm* and *Efs*, indicating genetic diversity among both species. However, when a “cluster” was defined as a set of STs that matched more than six of the seven loci, *Efs* strains were classified into 20 clusters while *Efm* were classified into seven clusters using the BURST algorithm. This suggests that the genome of *Efs* in our hospital may be more diverse and flexible than that of *Efm*. Briefly, *Efm* and *Efs*, which belong to the genus *Enterococcus*, showed significant differences in their drug resistance characteristics, distribution of virulence genes, clonality, and ability to form biofilms.

Nevertheless, many previous studies (Ghanem et al. 2007; Huh et al. 2023) on *Enterococcus* have included both *Efm* and *Efs*, particularly in relation to VRE. As vancomycin resistance is predominantly found in *Efm* and rarely in *Efs*, the inclusion of both species in the analysis would lead to significant collinearity between vancomycin resistance and species. Ignoring bacterial species may lead to biased analyses because of the different characteristics of pathogens (Kramer et al. 2018). Therefore, research on *Enterococcus* should be clearly defined whether the object of study is *Efm* or *Efs*; if both are studied, they should be analyzed as two different bacteria, not together.

In recent years, the ratio of *Efs* to *Efm* causing diseases has changed dramatically from 10:1 to 1:1, and even in our hospital, this ratio has been reversed to 1:1.6. We speculate that it may be because *Efm* is more adaptable to clinical settings where the antibiotic pressure is high and difficult to clear rapidly, leading to a continuous increase in *Efm*. This also indicates that, compared to being more prone to drug resistance, carrying more virulence genes and producing biofilms do not significantly increase the competitiveness of bacteria in hospitals.

As mentioned previously, there are many differences between *Efm* and *Efs*, but they share some common features. Both *Efm* and *Efs* BSI isolates enrolled for MLST analysis showed a polyclonal pattern with a dominant clone and many unique types, implying the coexistence of clonal dissemination and an influx of new clones. ST4 accounted for 29.2% of *Efs* and ST78 accounted for 46.6% of *Efm*, being the dominant clone in each and showing clonal dissemination. Other STs demonstrated a high degree of diversity and low population sizes, indicating that bacteria are rapidly evolving, constantly forming, and introducing new clones. Furthermore, the influx of new clones may occur not only between patients but also through the food chain. In this study, ST812 and ST94 in *Efm* were detected, which are genetically distantly related to other STs. These two STs have also been reported in *Efm* isolates from swine manure and feed samples (Zhao et al. 2022). These findings highlight the potential for foodborne transmission of *Enterococcus* clones. Therefore, it is important to continuously monitor the presence of *Enterococcus* clones and their potential sources of dissemination to ensure the effectiveness of preventive and control measures.

Honestly, the greatest clinical concern is the production and spread of VREfm. A decreasing trend (from 2.9% in 2014 to 1.2% in 2021) in the prevalence of VREfm was observed in the CARSS report. Other studies (Zhou et al. 2020) also reported the same tendency: resistance rates to vancomycin in *Efm* decreased annually from 9.3% in 2013 to 1.4% in 2018. However, this decreasing trend was not observed in this study.

Similar to other studies (Sun et al. 2019; Yan et al. 2023), molecular typing of VREfm by MLST revealed 12 STs, with one dominant clone (ST78, 41.7%) and some minor clones, indicating that both clonal expansion and horizontal transmission of resistance genes contribute to the prevalence of VRE in hospitals. However, the MLST data for VSEfm also showed a polyclonal pattern, with one dominant clone (ST78, 48.8%) and many specific types. Most STs contain both VREfm and VSEfm, and there is no clear evidence suggesting that a particular ST is associated with vancomycin resistance. Overall, we speculate that the horizontal transmission of resistance genes may play a more important role in the prevalence of VREfm than clonal expansion in our hospital.

By MLST analysis for *Efm*, over the 7-year study period, the dominant ST was always ST78, whether vancomycin resistant or not, whether biofilm forming or not. This is in line with other studies in which ST78 was already known to be prevalent in several European and Asian countries (Ryan et al. 2015). However, an Israeli study of 182 patients with VRE bacteremia showed that almost 50% of the isolates were ST772 and ST80 (Abu-Lybdeh et al. 2022). As of May 23, 2023, the 7485 *Efm* strains uploaded in the MLST database could be classified into 2283 ST types, with the top six STs ranked in the following order: ST17 (452, 6.0%), ST18 (390, 5.2%), ST78 (366, 4.8%), ST80 (364, 4.8%), ST117 (263, 3.5%), and ST203 (199, 2.6%), all of which belonged to CC17. These findings suggest that *Efm* isolates from our hospital do not have unique STs in their circulation to explain its high prevalence of VREfm.

Interestingly, the MLST data of *Efs* in this study showed that ST4 (21/72, 29.2%) was the most common, and was less frequently detected in other studies. A study from Saudi Arabia (Farman et al. 2019) noted a predominance of ST179 and ST16 in clinical *Efs* isolates, whereas another study from India (Rao et al. 2020) found a majority of ST28 and ST181 in multi-drug resistant *Efs* associated with high-risk hospital infections, and a study in Poland (Gawryszewska et al. 2021) of penicillin-resistant but ampicillin-susceptible *Efs* found an overwhelming predominance of ST6 strains (78/136, 57.4%). In China, the *Efs* strains in Shenzhen Province (Ma et al. 2021) are dominated by ST16 and ST179, which together account for more than 50% of the total. In the MLST database, and as of May 23, 2023, a total of 2938 *Efs* strains had been uploaded, with 1467 STs detected. The top six STs were ST6 (125, 4.2%), ST21 (101, 3.4%), ST16 (88, 2.9%), ST40 (73, 2.4%), ST179 (44, 1.4%), and ST82 (41, 1.3%). ST4 was ranked 23rd in the MLST database with only 15 (0.5%) of the uploaded strains. In fact, studies focusing on *Efs* bacteria are becoming less frequent because of the decreasing rate of clinical isolation and the decreasing prevalence of VRE each year.

Additionally, our results suggest that the distribution of virulence genes is not significantly related to biofilm formation, which is consistent with the findings of an Iranian study (Fallah et al. 2017). Another report from Egypt showed that *esp* + enterococcal isolates probably have strong/moderate biofilm-forming ability than *esp*- isolates, but no correlation between *gelE, asa1*, and *cylA* genes and biofilm-forming ability was observed (Aladarose et al. 2019). Several studies have investigated the role of virulence genes in biofilm formation. Some reports have suggested an association between some virulence factors and biofilm formation, whereas others have suggested that these genes are not necessary for biofilm formation. In conclusion, there is no consensus on the relationship between virulence factors and biofilm formation in *Efs* strains. The following factors may be considered when analyzing the reasons for this discrepancy: the complexity of biofilm formation, incomplete phenotypic and genotypic concordance of virulence factors, and strain and methodological differences.

This study had several limitations. First, this was a single-center study, and the findings may have been influenced by local epidemiological variables, thereby limiting their applicability to other settings. Second, this study only detected the presence of the *vanA* and *vanB* genes, excluding the *vanM* gene, which was first reported as a novel and prevalent glycopeptide resistance determinant in clinically relevant *Enterococcus* species in China. Recently, a multicenter study conducted in six hospitals in Beijing showed that the prevalence of *vanM* was 15.2% (Yan et al. 2023). However, whole-genome sequencing of 137 VRE strains isolated from our hospital revealed that only one VRE strain carried both *vanA* and *vanM* genes, while the remaining 136 strains harbored only the *vanA* gene, suggesting that *vanM* may not yet be endemic in our hospital (data not published). Third, only *Efm* and *Efs* bacteria isolated from patients with BSI were included; therefore, it may not provide a full picture of the molecular characteristics, but it may improve the clinical representation and significance.

## Conclusions

In summary, 116 *Efm* (36VRE + 80VSE) and 72 *Efs* (2VRE + 70VSE) isolated from patients with BSI over a 7-year study period in our hospital showed different drug resistance rates, diverse virulence gene patterns, varied biofilm formation capabilities, and various MLST types. The genomes of *Efm* and *Efs* both exhibited significant diversity and variability, which allowed for the widespread horizontal transmission of antibiotic resistance and virulence genes among species. The rapid evolution and recombination of the genome highlight the importance of actively monitoring the molecular features of bacteria.

### Supplementary information

Below is the link to the electronic supplementary material.

Supplementary file1 (DOCX 41 KB)

## Data Availability

All the data that support the findings of this study can be provided by the authors, upon reasonable request.
