# Supplementary material for: Molecular characterization and biofilm formation ability of Enterococcus faecium and Enterococcus faecalis bloodstream isolates from a Chinese tertiary hospital in Beijing
Source: Int Microbiol. 2023 Nov 6;27(3):929–39. doi: 10.1007/s10123-023-00441-2 (PMC11144123; doi:10.1007/s10123-023-00441-2)
Supplement: Supplementary file 1 — Supplementary file1 (DOCX 41 KB) [file 10123_2023_441_MOESM1_ESM.docx]

**Table S1 Profiles of 116 *Enterococcus faecium* isolates.**

| **Isolates** | **Date** | **Wards** | **Vancomycin^a^** | **MLST types** | **Virulence genes** | | **Biofilm^b^** |
| --- | --- | --- | --- | --- | --- | --- | --- |
|  |  |  |  |  | ***esp*** | ***hyl*** |  |
| efm1 | 2011/09 | Internal medicine | R | ST17 | + | + | - |
| efm2 | 2011/11 | Internal medicine | S | ST571 | + | - | - |
| efm3 | 2011/11 | Internal medicine | R | ST78 | + | - | + |
| efm4 | 2012/02 | Hematology | R | ST78 | + | + | - |
| efm5 | 2012/03 | Surgery | R | ST18 | - | + | - |
| efm6 | 2012/05 | Internal medicine | S | ST414 | + | - | - |
| efm7 | 2012/05 | Surgery | S | ST78 | + | - | + |
| efm8 | 2012/06 | Internal medicine | S | ST78 | + | + | - |
| efm9 | 2012/07 | Internal medicine | R | ST17 | + | + | - |
| efm10 | 2012/11 | Hematology | R | ST17 | - | - | - |
| efm11 | 2012/11 | Internal medicine | R | ST78 | + | - | + |
| efm12 | 2013/01 | Hematology | R | ST564 | + | + | - |
| efm13 | 2013/01 | Internal medicine | R | ST78 | + | - | - |
| efm14 | 2013/06 | Internal medicine | R | ST571 | + | - | - |
| efm15 | 2013/07 | Intensive care unit | R | ST78 | + | + | - |
| efm16 | 2013/09 | Surgery | S | ST78 | + | - | - |
| efm17 | 2013/09 | Emergency | S | ST78 | + | + | - |
| efm18 | 2013/10 | Internal medicine | R | ST571 | + | - | - |
| efm19 | 2013/10 | Internal medicine | R | ST78 | + | - | - |
| efm20 | 2013/10 | Surgery | S | ST32 | - | - | - |
| efm21 | 2013/10 | Internal medicine | S | ST192 | + | - | - |
| efm22 | 2013/10 | Internal medicine | S | ST18 | - | - | - |
| efm23 | 2013/11 | Internal medicine | R | ST78 | + | - | - |
| efm24 | 2013/11 | Internal medicine | S | ST812 | - | - | - |
| efm25 | 2013/12 | Surgery | S | ST78 | + | - | - |
| efm26 | 2014/01 | Surgery | S | ST78 | + | - | + |
| efm27 | 2014/01 | Hematology | S | ST78 | + | - | - |
| efm28 | 2014/02 | Internal medicine | S | ST17 | - | - | - |
| efm29 | 2014/02 | Internal medicine | S | ST78 | + | - | - |
| efm30 | 2014/05 | Hematology | S | ST262 | - | - | - |
| efm31 | 2014/07 | Internal medicine | R | ST78 | + | - | - |
| efm32 | 2014/07 | Hematology | S | ST252 | - | + | - |
| efm33 | 2014/07 | Internal medicine | S | ST78 | + | + | - |
| efm34 | 2014/08 | Internal medicine | R | ST18 | - | - | + |
| efm35 | 2014/08 | Internal medicine | S | ST78 | + | - | - |
| efm36 | 2014/08 | Internal medicine | S | ST78 | + | + | - |
| efm37 | 2014/08 | Surgery | S | ST78 | + | + | - |
| efm38 | 2014/08 | Internal medicine | S | ST78 | + | + | - |
| efm39 | 2014/09 | Internal medicine | R | ST78 | + | - | + |
| efm40 | 2014/10 | Intensive care unit | R | ST78 | + | - | - |
| efm41 | 2014/10 | Intensive care unit | R | ST17 | - | - | + |
| efm42 | 2014/11 | Internal medicine | R | ST78 | + | - | - |
| efm43 | 2014/11 | Intensive care unit | S | ST789 | - | - | - |
| efm44 | 2014/11 | Intensive care unit | S | ST341 | + | - | - |
| efm45 | 2014/11 | Internal medicine | S | ST812 | - | - | - |
| efm46 | 2014/12 | Internal medicine | S | ST78 | + | - | - |
| efm47 | 2014/12 | Surgery | S | ST202 | - | - | - |
| efm48 | 2014/12 | Internal medicine | S | ST78 | + | - | - |
| efm49 | 2015/01 | Hematology | S | ST78 | + | + | - |
| efm50 | 2015/02 | Internal medicine | S | ST78 | + | - | - |
| efm51 | 2015/03 | Internal medicine | R | ST78 | + | - | - |
| efm52 | 2015/04 | Surgery | S | ST218 | - | - | - |
| efm53 | 2015/08 | Internal medicine | S | ST78 | + | - | - |
| efm54 | 2015/09 | Surgery | S | ST18 | - | - | - |
| efm55 | 2015/10 | Internal medicine | S | ST78 | - | - | - |
| efm56 | 2015/11 | Hematology | S | ST80 | - | + | - |
| efm57 | 2015/11 | Internal medicine | S | ST17 | - | - | - |
| efm58 | 2015/11 | Internal medicine | S | ST812 | - | - | - |
| efm59 | 2015/12 | Intensive care unit | S | ST812 | - | - | - |
| efm60 | 2015/12 | Internal medicine | S | ST733 | + | - | - |
| efm61 | 2015/12 | Emergency | S | ST323 | + | + | - |
| efm62 | 2016/03 | Internal medicine | R | ST341 | + | - | + |
| efm63 | 2016/03 | Internal medicine | R | ST389 | + | + | - |
| efm64 | 2016/03 | Internal medicine | S | ST812 | - | - | - |
| efm65 | 2016/04 | Internal medicine | S | ST17 | - | - | - |
| efm66 | 2016/05 | Internal medicine | S | ST78 | + | - | + |
| efm67 | 2016/06 | Internal medicine | S | ST17 | + | + | - |
| efm68 | 2016/09 | Hematology | R | ST17 | + | + | - |
| efm69 | 2016/10 | Internal medicine | S | NT1 | - | - | - |
| efm70 | 2016/10 | Internal medicine | S | ST78 | + | - | - |
| efm71 | 2016/11 | Internal medicine | S | ST812 | - | - | - |
| efm72 | 2016/12 | Internal medicine | S | ST78 | + | - | - |
| efm73 | 2016/12 | Internal medicine | S | ST789 | + | - | - |
| efm74 | 2016/12 | Emergency | S | ST555 | - | - | + |
| efm75 | 2017/01 | Internal medicine | R | ST80 | + | - | - |
| efm76 | 2017/01 | Internal medicine | R | ST789 | + | + | - |
| efm77 | 2017/02 | Internal medicine | R | ST80 | + | - | - |
| efm78 | 2017/03 | Internal medicine | R | ST547 | + | + | - |
| efm79 | 2017/03 | Internal medicine | S | ST789 | + | + | + |
| efm80 | 2017/03 | Emergency | S | ST78 | + | - | - |
| efm81 | 2017/04 | Surgery | S | ST78 | + | - | - |
| efm82 | 2017/05 | Internal medicine | S | ST78 | + | - | - |
| efm83 | 2017/05 | Hematology | S | ST789 | - | + | - |
| efm84 | 2017/05 | Neurology | S | ST78 | + | - | + |
| efm85 | 2017/05 | Hematology | S | ST78 | + | - | - |
| efm86 | 2017/06 | Internal medicine | S | ST78 | + | - | + |
| efm87 | 2017/06 | Intensive care unit | S | ST78 | + | - | + |
| efm88 | 2017/07 | Intensive care unit | S | ST812 | - | - | + |
| efm89 | 2017/08 | Internal medicine | S | ST78 | + | - | + |
| efm90 | 2017/09 | Surgery | R | ST230 | + | + | + |
| efm91 | 2017/09 | Internal medicine | S | ST78 | + | - | - |
| efm92 | 2017/10 | Surgery | S | ST78 | + | - | + |
| efm93 | 2017/10 | Internal medicine | S | ST812 | - | - | - |
| efm94 | 2017/11 | Internal medicine | R | ST78 | + | - | - |
| efm95 | 2017/11 | Internal medicine | R | ST78 | + | + | - |
| efm96 | 2017/11 | Hematology | S | ST78 | + | - | - |
| efm97 | 2017/11 | Hematology | S | ST973 | + | + | - |
| efm98 | 2017/12 | Intensive care unit | R | ST192 | + | - | - |
| efm99 | 2017/12 | Internal medicine | R | ST789 | + | + | + |
| efm100 | 2017/12 | Internal medicine | S | ST78 | + | - | + |
| efm101 | 2017/12 | Emergency | S | ST78 | + | - | - |
| efm102 | 2017/12 | Internal medicine | S | ST733 | + | - | + |
| efm103 | 2018/01 | Neurology | R | ST78 | + | - | - |
| efm104 | 2018/01 | Surgery | R | ST80 | + | - | - |
| efm105 | 2018/01 | Intensive care unit | S | ST17 | + | - | - |
| efm106 | 2018/02 | Internal medicine | S | ST78 | + | - | - |
| efm107 | 2018/02 | Internal medicine | R | ST17 | + | - | - |
| efm108 | 2018/02 | Internal medicine | S | ST78 | + | - | - |
| efm109 | 2018/02 | Intensive care unit | S | NT2 | + | + | - |
| efm110 | 2018/02 | Internal medicine | S | ST78 | + | - | - |
| efm111 | 2018/02 | Internal medicine | S | ST78 | + | - | - |
| efm112 | 2018/02 | Surgery | S | ST94 | - | - | +++ |
| efm113 | 2018/02 | Internal medicine | S | ST547 | + | + | - |
| efm114 | 2018/02 | Hematology | S | ST547 | + | + | - |
| efm115 | 2018/03 | Internal medicine | S | ST812 | - | - | - |
| efm116 | 2018/03 | Internal medicine | S | ST262 | + | - | - |

^a^ R: vancomycin resistance; S: vancomycin susceptible.

^b^ +: weak biofilm; +++: strong bioflim; -: no biofilm.

**Table S2 Profiles of 72 *Enterococcus faecalis* isolates.**

| **Isolates** | **Date** | **Wards** | **Vancomycin^a^** | **MLST type** | **Virulence genes** | | | | **Biofilm^b^** |
| --- | --- | --- | --- | --- | --- | --- | --- | --- | --- |
|  |  |  |  |  | ***esp*** | ***gelE*** | ***asa*** | ***cylA*** |  |
| efa1 | 2011/07 | Internal medicine | S | ST21 | + | + | + | + | ++ |
| efa2 | 2011/08 | Internal medicine | R | ST4 | + | + | + | + | ++ |
| efa3 | 2011/10 | Surgery | S | ST6 | + | + | + | + | ++ |
| efa4 | 2011/11 | Neurology | S | NT8 | + | + | + | + | ++ |
| efa5 | 2012/01 | Internal medicine | S | ST21 | - | + | - | - | ++ |
| efa6 | 2012/03 | Surgery | S | ST116 | - | + | + | - | - |
| efa7 | 2012/09 | Surgery | S | ST4 | + | + | + | + | ++ |
| efa8 | 2012/05 | Internal medicine | S | ST6 | + | + | + | + | +++ |
| efa9 | 2012/12 | Intensive care unit | S | NT4 | + | + | + | + | +++ |
| efa10 | 2012/09 | Neurology | R | ST4 | + | + | + | + | + |
| efa11 | 2012/12 | Surgery | S | ST21 | - | + | - | - | +++ |
| efa12 | 2012/05 | Surgery | S | ST4 | + | + | + | + | + |
| efa13 | 2013/04 | Internal medicine | S | ST4 | - | + | + | + | ++ |
| efa14 | 2013/02 | Surgery | S | ST4 | + | + | + | + | + |
| efa15 | 2013/02 | Neurology | S | ST6 | - | + | + | + | +++ |
| efa16 | 2013/02 | Internal medicine | S | NT8 | - | - | + | - | + |
| efa17 | 2013/07 | Internal medicine | S | ST179 | + | + | + | + | +++ |
| efa18 | 2013/08 | Internal medicine | S | ST25 | + | + | - | - | + |
| efa19 | 2013/08 | Internal medicine | S | ST4 | + | + | + | + | ++ |
| efa20 | 2013/09 | Internal medicine | S | ST721 | - | + | + | - | + |
| efa21 | 2014/01 | Internal medicine | S | ST16 | + | - | + | + | ++ |
| efa22 | 2014/04 | Surgery | S | ST16 | + | - | + | + | ++ |
| efa23 | 2014/07 | Internal medicine | S | ST16 | + | - | + | + | ++ |
| efa24 | 2014/08 | Internal medicine | S | ST179 | + | + | + | + | + |
| efa25 | 2014/08 | Intensive care unit | S | NT9 | + | + | + | + | - |
| efa26 | 2014/08 | Internal medicine | S | ST16 | + | - | + | + | + |
| efa27 | 2014/09 | Surgery | S | ST16 | + | + | + | + | ++ |
| efa28 | 2014/09 | Intensive care unit | S | ST16 | + | + | + | + | ++ |
| efa29 | 2014/01 | Neurology | S | ST4 | - | + | + | - | +++ |
| efa30 | 2014/04 | Neurology | S | ST179 | + | + | + | + | ++ |
| efa31 | 2014/01 | Internal medicine | S | ST4 | + | + | + | + | ++ |
| efa32 | 2015/02 | Internal medicine | S | ST4 | + | + | + | + | ++ |
| efa33 | 2015/05 | Intensive care unit | S | NT1 | - | + | - | - | +++ |
| efa34 | 2015/08 | Neurology | S | ST4 | + | + | + | + | ++ |
| efa35 | 2015/10 | Internal medicine | S | ST480 | + | - | + | - | + |
| efa36 | 2015/12 | Surgery | S | ST179 | + | + | + | + | +++ |
| efa37 | 2015/12 | Neurology | S | ST6 | - | + | + | - | +++ |
| efa38 | 2016/01 | Internal medicine | S | ST506 | - | + | - | - | +++ |
| efa39 | 2016/02 | Surgery | S | NT3 | + | + | + | + | ++ |
| efa40 | 2016/02 | Surgery | S | ST4 | - | + | + | + | +++ |
| efa41 | 2016/03 | Internal medicine | S | ST28 | - | + | + | + | - |
| efa42 | 2016/03 | Internal medicine | S | ST126 | - | + | - | - | + |
| efa43 | 2016/03 | Surgery | S | NT5 | + | + | + | + | ++ |
| efa44 | 2016/03 | Internal medicine | S | ST126 | - | + | - | - | + |
| efa45 | 2016/04 | Internal medicine | S | NT10 | + | + | + | + | ++ |
| efa46 | 2016/04 | Emergency | S | ST207 | - | + | - | - | - |
| efa47 | 2016/04 | Neurology | S | ST4 | + | + | + | + | ++ |
| efa48 | 2016/05 | Neurology | S | ST4 | + | + | + | + | +++ |
| efa49 | 2016/07 | Intensive care unit | S | ST179 | + | + | + | + | ++ |
| efa50 | 2016/08 | Surgery | S | ST16 | - | - | + | + | ++ |
| efa51 | 2016/08 | Internal medicine | S | ST16 | + | - | + | + | ++ |
| efa52 | 2016/10 | Neurology | S | ST632 | + | + | + | + | + |
| efa53 | 2016/10 | Gynecology and obstetrics | S | ST65 | - | + | + | - | ++ |
| efa54 | 2016/10 | Internal medicine | S | ST4 | + | + | + | + | ++ |
| efa55 | 2016/10 | Gynecology and obstetrics | S | ST59 | - | + | - | - | ++ |
| efa56 | 2016/11 | Internal medicine | S | ST4 | + | + | + | + | ++ |
| efa57 | 2016/12 | Internal medicine | S | ST4 | + | + | + | + | ++ |
| efa58 | 2016/12 | Emergency | S | ST28 | - | + | + | + | - |
| efa59 | 2017/05 | Internal medicine | S | ST28 | - | + | + | + | + |
| efa60 | 2017/05 | Neurology | S | ST6 | - | + | + | - | +++ |
| efa61 | 2017/05 | Neurology | S | ST4 | + | + | + | + | ++ |
| efa62 | 2017/08 | Intensive care unit | S | NT6 | - | + | - | - | +++ |
| efa63 | 2017/08 | Internal medicine | S | ST4 | + | - | + | + | +++ |
| efa64 | 2017/11 | Internal medicine | S | ST4 | + | + | + | + | + |
| efa65 | 2017/11 | Neurology | S | ST6 | - | + | + | - | +++ |
| efa66 | 2017/12 | Internal medicine | S | ST6 | - | + | + | - | +++ |
| efa67 | 2017/12 | Surgery | S | ST116 | + | + | + | + | +++ |
| efa68 | 2017/12 | Internal medicine | S | ST28 | - | + | + | + | + |
| efa69 | 2018/02 | Intensive care unit | S | NT2 | + | - | + | + | ++ |
| efa70 | 2017/08 | Internal medicine | S | NT7 | + | - | - | - | +++ |
| efa71 | 2017/09 | Gynecology and obstetrics | S | ST179 | + | + | + | + | - |
| efa72 | 2017/05 | Internal medicine | S | ST4 | - | + | + | - | +++ |

^a^ R: vancomycin resistance; S: vancomycin susceptible.

^b^ +: weak biofilm; ++: moderate biofilm; +++: strong bioflim; -: no biofilm.
